# Sex Chromosomes Do It Differently

**DOI:** 10.1371/journal.pbio.2001096

**Published:** 2016-10-13

**Authors:** Lauren A. Richardson

**Affiliations:** Public Library of Science, San Francisco, California, United States of America

**Fig 1:**
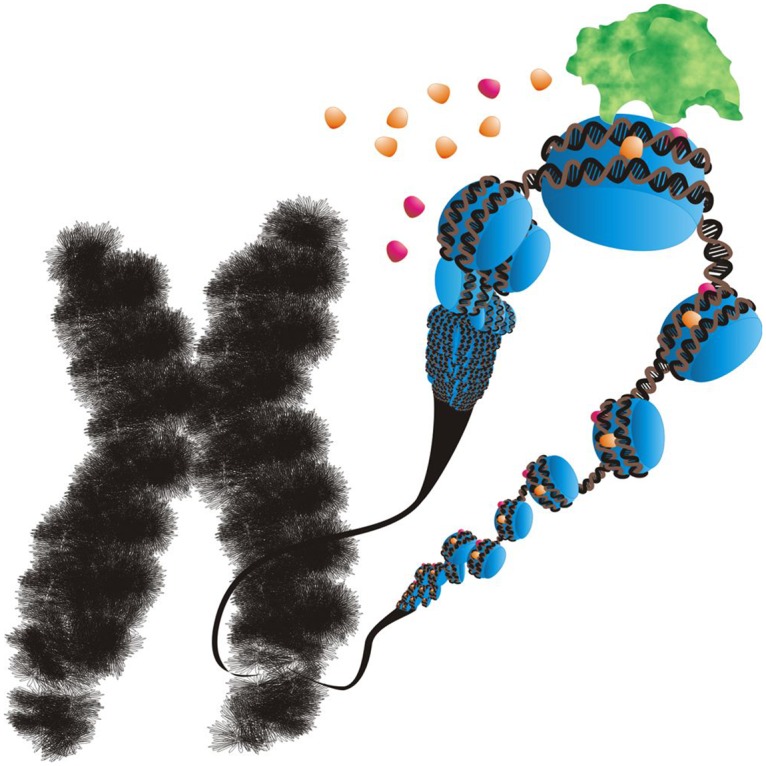
Transcribing the X Chromosome. Regulating gene transcription from the sex chromosomes requires many distinct mechanisms. *Image Credit*: *Image by Dr*. *Marian L*. *Miller*.

Organisms are finely tuned systems that are resilient to perturbations but that must maintain some constants. For example, within the genome, the ratio between different gene products must be tightly constrained to ensure stoichiometric assembly of protein complexes and the like. Evolution has honed expression levels so that as long as there is the same number of chromosomes per cell, the relative transcription levels of such genes can be kept constant (Fig 1).

But what happens if the ratio of chromosomes varies between individuals? This is precisely the case with heterogametic sex chromosomes (such as the human XY and XX chromosomes), which are common and have evolved independently from ancestral autosomes (non-sex chromosomes) several times in plants and animals. The evolutionary loss of recombination between X and Y chromosomes has led to distinct differences in gene content, and the resulting disparity in chromosome (and therefore gene) dosage between the sexes creates a number of challenges.

Mammals solve this conundrum by inactivating one of the two X chromosomes in each female somatic cell. Flies, which also have X and Y sex chromosomes, albeit independently evolved, do things differently—they upregulate the single X chromosome in male somatic cells. The overall consequence, however, in both mammals and flies, is chromosomal dosage compensation. By contrast, in male germline cells, both the X and Y are transcriptionally repressed in a process known as meiotic sex chromosome inactivation (MSCI), for reasons that remain somewhat unclear.

While MSCI leads to a significant decrease in gene expression from the germline X chromosome, there are genes on the X chromosome that must be expressed for successful spermatogenesis. A recent paper published in PLOS Biology has revealed new insights into the transcriptional repression of the X chromosome in the *Drosophila* male germline [1]. The mechanism they describe is distinct from classic mammalian MSCI, and they refer to it as X chromosome suppression. They find that most genes on the male germline X chromosome are transcriptionally suppressed about 2–4 fold compared to their expression in somatic cells. The testis-specific genes on the X chromosome, however, seem to escape this suppression, apparently by evolving very strong testis-specific promoters in a gene-by-gene manner that are able to overcome the transcriptional suppression. Thus, evolutionary tug-of-war has achieved a balance between X chromosome-wide transcriptional suppression and the need for expression of testis-specific genes.

Mechanisms of MSCI vary between organisms, and in mice, most or all protein-coding genes on the X chromosome are silenced during MSCI. A study in *PLOS Genetics* shows that in addition to silencing protein-coding genes, MSCI represses the transcription of X-linked non-coding RNAs, including microRNAs with testis-specific expression [2]. Using single-cell RNA FISH, they find that the repression of these X-linked miRNA genes is essential for spermatogenesis, with forced expression causing spermatogenic defects.

Humans, like mice, exhibit strong MSCI in the male germ line, but it appears that this was a relatively recent evolutionary event; a paper in *BMC Biology* presents evidence that the platypus uses a transcriptional repression regime more similar to birds than the rest of the therian mammals (marsupials and placental mammals) [3]. Thus, rather than bearing the marks of full repression, platypus sex chromosomes have a general low level of transcription, suggesting that classic mammalian MSCI evolved after the divergence of monotremes.

While studying the distinct gene content of the mammalian X chromosome, authors of a *PLOS Biology* study noticed an interesting trend: genes residing on the X chromosome are disproportionately lowly expressed. They found that genes on the X chromosome have maximal expression levels about three times lower than that of autosomal genes, have a lower breadth of expression, and include fewer genes expressed in tissues requiring high levels of transcription (such as the liver) [4]. The authors reason that this is to avoid the transcriptional “traffic jams” that would arise from the combination of highly expressed genes and the evolutionary upregulation needed to compensate for the lower X chromosome dosage compared with the autosomes.

An essential step in establishing dosage compensation is to single out the X chromosome from the autosomes for this transcriptional activation. Work appearing in *PLOS Genetics* reveals how the X chromosome is identified in dipteran flies [5], which have an incredible diversity of sex chromosome configurations [6]. By comparing these sex chromosomes, the authors found that newly evolved X chromosomes acquire dosage compensation via the expansion of GA dinucleotide repeats, which can be generated by slippage of DNA polymerase. These repeats are then bound by the CLAMP zinc finger protein, which in turn recruits the dosage compensation complex to the X chromosome.

In *Drosophila*, some components of the dosage compensation machinery are also involved in the transcriptional activation of genes within heterochromatin on autosomes. The authors of a *PLOS ONE* paper identify those components of the dosage compensation machinery that are required for this heterochromatic gene expression [7]. Interestingly, the requirement for these components in heterochromatin is male-specific, which the authors posit may contribute to the observed sex-biased differences in heterochromatin gene expression.

Sex-biased genes are those that have a higher transcript level in one gender compared to the other. A study in *PLOS Genetics* investigated whether there were different patterns of sex-biased expression in early *Drosophila* embryos [8]. At this stage the genetic control shifts from the mother—who contributes mRNA at fertilization—to the zygote, and the authors explored how gene expression differs in early female and male embryos. Unlike the pervasive male bias in gene expression that is seen in adult flies, in embryos they see extensive female bias. They attribute this shift to the delay in establishing the dosage compensation machinery on the male X chromosome.

One can think of dosage compensation as preventing sex bias, but it can also lead to sexual conflict. If a mutation leads to increased expression in the heterogametic sex (e.g., XY) to balance expression, this could lead to hyperexpression in the homogametic sex (e.g., XX), with negative fitness consequences. In birds, females are the heterogametic sex (with ZW chromosomes) and males are homogametic (ZZ) and—unlike flies, worms, and mammals—birds have incomplete dosage compensation. To understand why this less efficient mechanism evolved, a *Nature Communications* study used a population genetic model and chicken transcriptomic data [9] to show that sexual conflict can account for the differences in dosage compensation seen between XY and ZW species; indeed their work suggests that sexual selection was critical for the evolution of chromosome dosage compensation.

There are still many outstanding mysteries of sex chromosome transcriptional regulation, many of which stem from the extensive variation between organisms. Even closely related species show distinct differences. For example, in *Drosophila melanogaster*, a gene inserted into the X chromosome will be dosage compensated. However, as shown in this *PLOS ONE* paper, in the Australian sheep blowfly *Lucilia cuprina*, inserted genes are not dosage compensated even though the endogenous genes are [10]. How dosage compensation between these two fly species differs is still unknown.

For more detailed reading please see the associated PLOS Collection [11].
